# Modality matters for the expression of inducible defenses: introducing a concept of predator modality

**DOI:** 10.1186/1741-7007-11-113

**Published:** 2013-11-18

**Authors:** Quirin Herzog, Christian Laforsch

**Affiliations:** 1 Department of Biology II, Ludwig-Maximilians-University Munich, Großhadernerstr. 2, Planegg-Martinsried 82152, Germany; 2 Department of Animal Ecology I, University of Bayreuth, Universitätsstr. 30, Bayreuth 95440, Germany

## Abstract

**Background:**

Inducible defenses are a common and widespread form of phenotypic plasticity. A fundamental factor driving their evolution is an unpredictable and heterogeneous predation pressure. This heterogeneity is often used synonymously to quantitative changes in predation risk, depending on the abundance and impact of predators. However, differences in ‘modality’, that is, the qualitative aspect of natural selection caused by predators, can also cause heterogeneity. For instance, predators of the small planktonic crustacean *Daphnia* have been divided into two functional groups of predators: vertebrates and invertebrates. Predators of both groups are known to cause different defenses, yet predators of the same group are considered to cause similar responses. In our study we question that thought and address the issue of how multiple predators affect the expression and evolution of inducible defenses.

**Results:**

We exposed *D. barbata* to chemical cues released by *Triops cancriformis* and *Notonecta glauca*, respectively. We found for the first time that two invertebrate predators induce different shapes of the same morphological defensive traits in *Daphnia*, rather than showing gradual or opposing reaction norms. Additionally, we investigated the adaptive value of those defenses in direct predation trials, pairing each morphotype (non-induced, *Triops*-induced, *Notonecta*-induced) against the other two and exposed them to one of the two predators. Interestingly, against *Triops*, both induced morphotypes offered equal protection. To explain this paradox we introduce a ‘concept of modality’ in multipredator regimes. Our concept categorizes two-predator-prey systems into three major groups (functionally equivalent, functionally inverse and functionally diverse). Furthermore, the concept includes optimal responses and costs of maladaptions of prey phenotypes in environments where both predators co-occur or where they alternate.

**Conclusion:**

With *D. barbata*, we introduce a new multipredator-prey system with a wide array of morphological inducible defenses. Based on a ‘concept of modality’, we give possible explanations how evolution can favor specialized defenses over a general defense. Additionally, our concept not only helps to classify different multipredator-systems, but also stresses the significance of costs of phenotype-environment mismatching in addition to classic ‘costs of plasticity’. With that, we suggest that ‘modality’ matters as an important factor in understanding and explaining the evolution of inducible defenses.

## Background

Predation is a strong selective force which drives evolution of prey defenses. Due to its variable nature, it is known to cause adaptations in the form of plastic responses in phenotypes, termed inducible defenses. Since they were first described [1] extensive research has revealed that this phenomenon is extremely widespread in many taxa, including bacteria [2], plants [3-5], invertebrates [6] and vertebrates [7,8]. For inducible defenses to evolve, four prerequisites have to be met: (I) the ability to form effective defenses, (II) associated costs that can offset the benefit in times with no or low predation, depending on the environmental conditions, (III) reliable cues to assess the current state of predation and (IV) heterogeneity of predation impact [9]. To date heterogeneity has often been used synonymously with variation in predation intensity (that is, the quantity of prey consumed or density of predators), caused by the presence or absence of predators (for example, by seasonal patterns [10]). However, it is not only relevant how much prey is eaten. It is also of importance which predator consumes the prey. It is known that different predators often pose different threats to their prey [11] and that predators can change their impact throughout their own [12] or their prey’s ontogeny [13]. Thus, the specific modality (that is, the qualitative aspect of natural selection caused by predation) also plays an important role. Modality describes where natural selection is leading in terms of direction and magnitude. Differences in this modality can result from a variety of entangled ecological factors, such as prey-preference, feeding mechanism, predation strategy, habitat use, dangerousness and the mode of perception of the predator [14]. In contrast to predation intensity, measuring, characterizing and comparing modality is difficult, even more so without clear categories and definitions. Additionally, variation in intensity and modality are non-exclusive changes, which can occur both on a spatial and a temporal scale, further complicating an assessment. Since most studies concentrate on single predator systems, modality differences have been largely neglected. However, as Sih *et al*. [15] pointed out, almost all prey organisms have to face multiple predators. Under these circumstances, modality matters. Indeed, many studies on amphibians [7,8,16,17], mollusks [11,18-20], insects [21], rotifers [22,23] and crustaceans [6,24] have demonstrated predator-specific responses, emphasizing the importance of modality.

*Daphnia*, a group of model organisms in ecology, evolution and biomedical research [25,26], provide a classical example for the role of modality. The predators they are facing are commonly categorized as invertebrate and vertebrate predators [27]. While vertebrate predators are considered to be primarily visual hunters and prefer larger prey, invertebrates are generally regarded as size-limited and mostly tactile predators. Corresponding to these different modalities, the well-known responses of daphnids exposed to fish are to reproduce earlier at a smaller size [28,29], to release more but smaller offspring [28] and to migrate into darker and deeper water layers during the day [30,31]. In contrast, when encountering invertebrate predators, such as *Chaoborus* larvae, daphnids mature later at larger size and produce fewer but larger offspring [28,29,32]. These above mentioned changes are, however, restricted to life history and behavioral defenses, with especially the latter considered to adapt fast and reversibly [33,34]. Yet, more prominent features of the genus *Daphnia* are numerous plastic morphological responses, such as helmets [35], crests [36], neckteeth [37,38], elongated tail-spines [13,39] and a crown of thorns [40]. Except in one species (*Daphnia lumholtzi*[39]), these defenses are solely built against invertebrate predators. While in one case they indeed have been shown to be caused by and act against multiple invertebrate predators [41], in most cases they seem to be predator specific [36,37,39,40,42]. Although this clearly questions the grouping of ‘invertebrate predators’ together as a single functional group, the potential differences in their modality have not been the focus of research so far.

In this context, we investigated if differences in the modality of invertebrate predators are relevant for the expression of inducible defenses. We used two contrasting predators with distinct differences in their morphology and ecology (that is, predation strategy): *Triops cancriformis* (Notostraca) and *Notonecta glauca* (Hemiptera). In addition, both predators are known to induce morphological defenses in *Daphnia*[13,36,40,43]. As the prey organism, we used a clone of *Daphnia barbata*, an African pond and lake dwelling species [44], which shares distribution and habitats with predators of both genera [45-47]*.* As a first step, we exposed *D. barbata* to the chemical cues released from both predators separately and analyzed morphological responses among all experimental groups. As a second step, we used direct predation trials to assess the adaptive value of each morphotype. We show that two invertebrate predators can induce different morphological defensive traits in *D. barbata*, which are based on the same structures, but built in different shapes. This is not only the first record of inducible defenses in *D. barbata*, but a unique case of defensive specialization across a wide range of taxa. Surprisingly, the defense against one predator also offered protection against the other predator, in one case even matching the specialized defense. To explain why the prey shows nonetheless not one general but two distinctively defended morphotypes, a theoretical framework is needed. Therefore, we introduce a ‘concept of modality' , which categorizes multipredator-prey systems into three major groups (functionally equivalent, functionally inverse and functionally diverse) and describes optimal responses in environments where predators co-occur or alternate. This concept is in line with the existing literature, but provides a general framework. It offers an explanation for the evolution of the different induced morphotypes of *D. barbata*, generates a basis to assess and compare the importance of modality in different multi-predator-prey systems and emphasizes the importance of a differentiation between predator co-occurrence and predator succession.

## Results

### Morphological parameters

**Figure 1:**
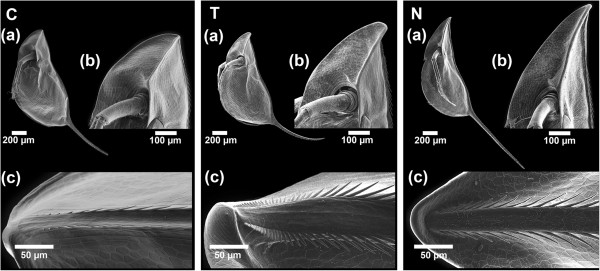
**The morphotypes of *D. barbata.*** SEM pictures, showing the control morph **C**, the *Triops*-induced morph **T** and the *Notonectid*-induced morph **N** from a lateral view **(a)**, a detailed view of the helmet **(b)** and the dorsal ridge at the top of the helmet **(c)**.

Significant changes in the morphology of *D. barbata* (Figure 1) between the treatments and within all measured parameters were observed (Kruskal-Wallis one-way analysis of variance, all *P* <0.001). Relative helmet length was significantly different in all three treatments (all pair wise comparisons *P* ≤0.001; Table 1). The control (non-predator exposed) daphnids had the smallest helmets. Larger helmets were found in the *Triops*-induced treatment and the longest helmets overall were from *Notonecta*-exposed daphnids (Table 1, Figure 1). The shape of the helmet varied as well. Daphnids exposed to *T. cancriformis* built a backwards bending helmet which differs significantly in its angle relative to the body axis from both the control (*P* <0.001; Table 1) and *Notonecta*-induced daphnids (*P* <0.001).

**Table 1:** Measured morphological parameters

				**Kruskal-Wallis main test**	**Kruskal-Wallis pairwise comparison**		
**Parameters**	**Group**	**Mean**	**SEM**			**H**	***P*-value**
**Helmet**							
**Relative helmet length (helmet length/body length)**	**C** (n = 17)	0.260	0.004	df = 2	**C - N**	−37.765	<0.001
	**N** (n = 17)	0.384	0.008	H = 47.235	**C - T**	−19.190	0.001
	**T** (n = 21)	0.322	0.004	*P* = 0.001	**N - T**	18.574	0.001
**Helmet angle [°]**	**C** (n = 17)	110.320	0.897	df = 2	**C - N**	−11.706	0.099
	**N** (n = 17)	115.463	0.955	H = 38.662	**C - T**	20.106	<0.001
	**T** (n = 21)	103.573	0.559	*P* = 0.001	**N - T**	31.812	<0.001
**Tail-spine**							
**Relative tail-spine length (tail-spine length/body length)**	**C** (n = 17)	0.583	0.014	df = 2	**C - N**	−26.529	<0.001
	**N** (n = 17)	0.859	0.019	H = 34.720	**C - T**	1.756	1
	**T** (n = 21)	0.581	0.008	*P* = 0.001	**N - T**	28.286	<0.001
**Spine angle (°)**	**C** (n = 17)	160.518	1.264	df = 2	**C - N**	−3.471	1
	**N** (n = 17)	162.494	0.811	H = 38.222	**C - T**	25.61	<0.001
	**T** (n = 21)	143.596	0.844	*P* = 0.001	**N - T**	29.081	<0.001
**Curvature (absolute/effective spine length)**	**C** (n = 17)	1.005	0.001	df = 2	**C - N**	5.529	0.943
	**N** (n = 17)	1.003	0.000	H = 34.493	**C - T**	−22.964	<0.001
	**T** (n = 21)	1.018	0.001	*P* = 0.001	**N - T**	−28.493	<0.001
**Dorsal ridge**							
**Dorsal ridge width (μm)**	**C** (n = 17)	30.391	0.554	df = 2	**C - N**	0.294	1
	**N** (n = 17)	29.857	0.676	H = 37.094	**C - T**	−26.853	<0.001
	**T** (n = 21)	50.289	0.905	*P* = 0.001	**N - T**	−27.147	<0.001
**Dist. 1. to 10. microspine (μm)**	**C** (n = 17)	192.558	3.070	df = 2	**C - N**	17.000	0.005
	**N** (n = 17)	134.432	3.981	H = 47.016	**C - T**	35.500	<0.001
	**T** (n = 21)	47.235	1.293	*P* = 0.001	**N - T**	18.500	0.001
**Max. microspine length (μm)**	**C** (n = 17)	39.181	1.690	df = 2	**C - N**	−33.706	<0.001
	**N** (n = 17)	61.260	1.283	H = 39.885	**C - T**	−20.982	<0.001
	**T** (n = 21)	54.249	1.004	*P* = 0.001	**N - T**	12.724	0.043
**Microspine angle (°)**	**C** (n = 17)	19.533	0.906	df = 2	**C - N**	−13.941	0.029
	**N** (n = 17)	28.490	1.243	H = 43.776	**C - T**	−33.971	<0.001
	**T** (n = 21)	78.571	1.307	*P* = 0.001	**N - T**	−20.029	<0.001

C, non-induced daphnids (control); N, *Notonecta-*induced daphnids; T, *Triops*-induced daphnids; SEM, standard error of mean; H, test statistics.

Furthermore, the length of the tail-spine increased significantly with exposure to *Notonecta* as compared to both the control (*P* <0.001; Table 1) and *Triops-*induced daphnids (*P* <0.001; Table 1). *D. barbata* exposed to *Triops* did not increase tail-spine length compared to the control, but the morphology of the tail-spine was altered. Specifically, the tail-spine was bent backwards (lower spine angle) and had significantly more curvature as compared to the two other treatments (*P* <0.001; Table 1).

*Triops*-induced *D. barbata* showed an increase in microspine density at the cranial dorsal ridge (distance between 1st and 10th microspine; Table 1), a widening of the dorsal ridge, longer microspines and a sideways orientation of the 5th microspine (all *P* <0.001 compared to control; Table 1). *D. barbata* exposed to chemical cues released by *Notonecta* on the other hand showed a much smaller decrease in the distance between 1st and 10th microspine (*P* = 0.001; Table 1) and no changes in the dorsal ridge width (*P* = 1; Table 1). Additionally, they possessed longer microspines than *Triops*-induced daphnids (*P* = 0.043; Table 1) and compared to the control showed only a minor increase in the angle of the fifth microspine relative to the dorsal ridge (*P* <0.001; Table 1).

### Predation trials

**Figure 2:**
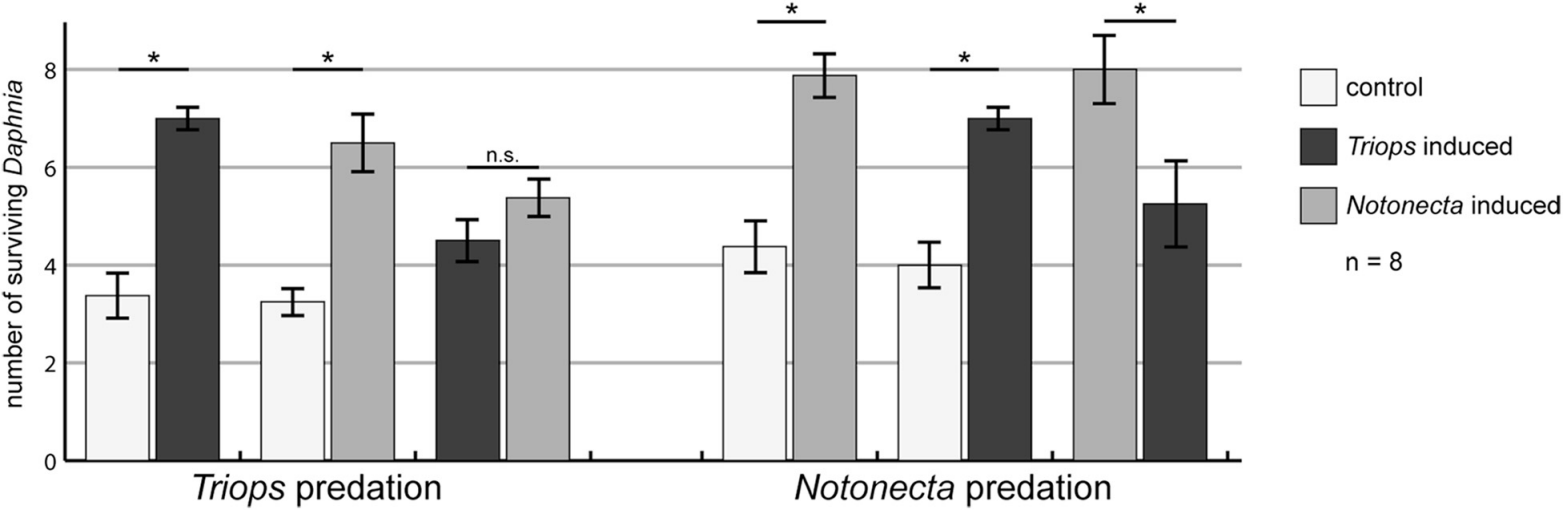
**Comparison of numbers of surviving primiparous daphnids in the predation trials.** Each of the three treatments was paired against the others as indicated by the strokes on the x-axis. The left side shows predation trials conducted with *T. cancriformis* as the predator and the right side shows predation trials where *N. glauca* served as predator. The error bars indicate standard error of mean. Asterisks indicate statistically significant results; n.s., not significant.

Predation trials using *Notonecta* revealed that the *Notonecta*-induced morphotype is better protected, having an 80% higher survivorship compared to the control (Wilcoxon signed-rank test, *P* = 0.012, Figure 2). The *Triops*-induced morphotype also held an advantage, having a 52% higher survivorship compared to the control (Wilcoxon signed-rank test, *P* = 0.028). However, the defenses proved to be less effective against notonectids in direct comparison with the *Notonecta*-induced morphotype (Wilcoxon signed-rank test, *P* = 0.017). In contrast, when *T. cancriformis* was the predator, both morphs showed higher survival rates compared to the control (107% increase for the *Triops*-induced morphotype, Wilcoxon signed-rank test, *P* = 0.017; 100% increase for the *Notonecta*-induced morphotype, Wilcoxon signed-rank test, *P* = 0.018). Between the two induced morphs, no significant differences in the number of surviving *Daphnia* were found (*P* = 0.230).

## Discussion

**Figure 3:**
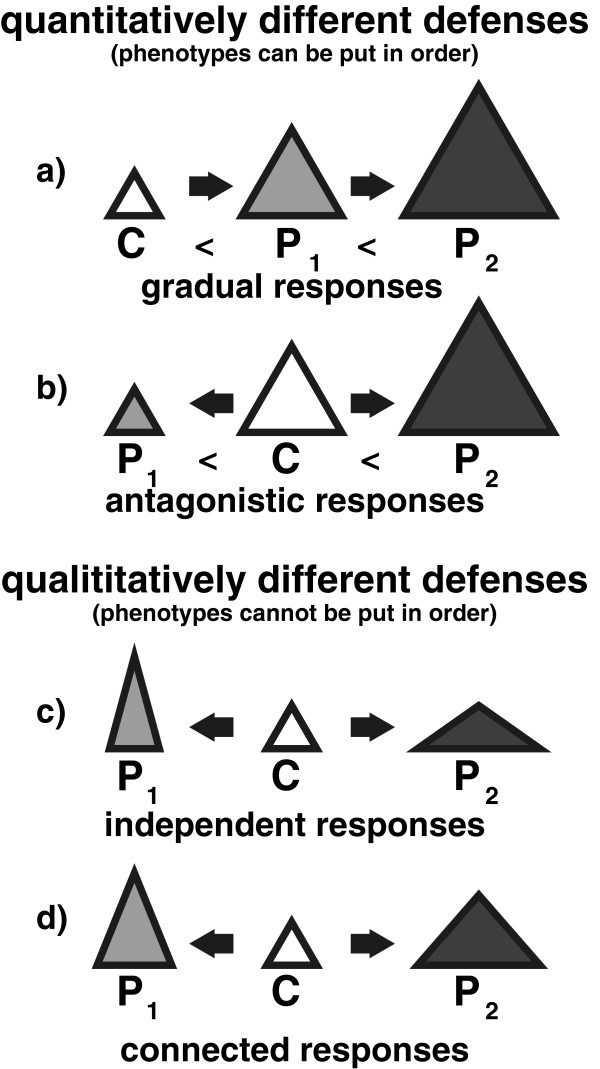
**Distinction between quantitative (a, b) and qualitative differences (c, d) of inducible defenses. C** (white) represents a non-induced morph, **P**_**1**_ (light gray) represents a morph defended against the predator 1 and **P**_**2**_ (dark gray) represents a morph defended against predator 2. The triangles, the square and the circle depict the phenotype. In the case of quantitative differences, the *changes can be put in order* in terms of an increase or decrease (represented by the different **sizes** of the triangles). This is true for both **a)** gradual responses (**C** <**P**_**1**_ <**P**_**2**_) and **b)** antagonistic responses (**P**_**1**_ <**C** <**P**_**2**_) In contrast, qualitative differences cannot be put in order in terms of an increase or decrease (represented by the different shapes of the triangles), as changes in different traits would lead to differently shaped phenotypes. This can either be the case, because **a)** independent changes occur (here: **P**_**1**_ gets higher than **C** and **P**_**2**_ gets wider than **C,** so for one trait (for example, width) it is **C** = **P**_**1**_ <**P**_**2**_ for the other trait (for example, height) it is **C** = **P**_**2**_ <**P**_**1**_), or **b)** because the changes to the traits occur to a different extent (here: **P**_**1**_ is higher than **P**_**2**_, but **P**_**2**_ is wider than **P**_**1**_, so for one trait (for example, width) it is **C** <**P**_**1**_ <**P**_**2**_ for the other trait (for example, height) it is **C** <**P**_**2**_ <**P**_**1**_).

Our findings are the first records of inducible defenses in *D. barbata*. Furthermore, we show that *D. barbata* responds to two different invertebrate predators (*Notonecta* and *Triops*) with distinctive morphological responses, rather than displaying a general defense. Unlike in previous records of predator-specific morphological responses across wide taxonomical groups, they consist of neither a gradual extension of the same trait (that is, an intermediate response against one predator and a stronger response against the other predator for example, [24,36]), nor of opposing traits (that is, when a trait increases against one predator and decreases against the other predator compared to the non-induced morph for example, [11,48,49]) or the addition of a new trait (for example, a high-tail against one predator and a high tail and a bulgy head against another [7]). Instead, the defenses are based on the same structures, but formed in a different way. This makes it impossible to order the morphotypes of *D. barbata* by the magnitude of expression of their traits (that is, quantitative differences, see Figure 3). Rather, the differences represent distinctive shapes, providing a rare example of qualitative predator specific defenses (see Figure 3, in accordance with Bourdeau [20]).

**Figure 4:**
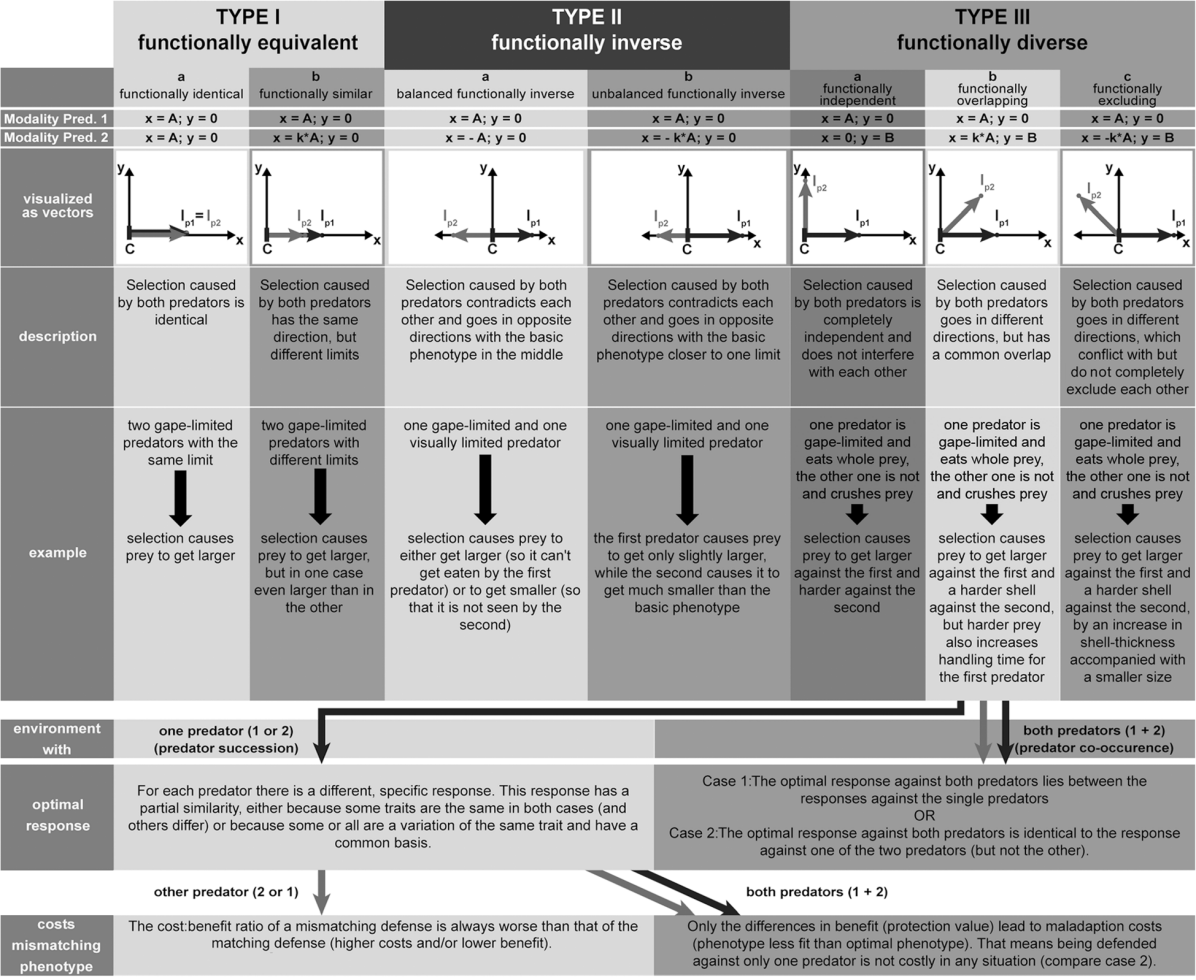
**Concept for the role of modality in systems with two predators.** The upper section describes the three basic types of modality differences with their subgroups (**a**) *sensu strictu*, **b**) *and***c**) *sensu latu*). To visualize modality (that is, the qualitative aspect of selection pressure caused by predation) two points are needed. The basic phenotype (that is, the phenotype in an environment without any predation pressure) serves as the initial point **C**, lying on the origin. The ‘immunity point’ **I**_**px**_ represents the terminal point, after which natural selection caused by predator x stops (that is, the phenotype is completely defended or ‘immune’). Its coordinates are defined by the modality of the predators given in the first and second row (‘Modality pred. 1’, colored black,’ Modality pred. 2’ colored gray) with **k** being a positive coefficient and **A**/**B** as variables. Between **C** and **I**_**px**_ a vector can be formed, representing the direction and length of selection. In the case of predator 1, this vector always lies on the x-axis; therefore, the protection of a phenotype against predator 1 can be read off its x-coordinate. The same is true for predator 2 in type I and II systems, but not for type III. For each type, a description and a theoretical example are given. Additionally for type IIIb, optimal responses in environments with a single (left) or both (right) predators as well as the costs for a mismatching phenotype (defended against the wrong or only one predator) are described in the bottom boxes.

Regarding the adaptive value of these differing traits, the morphs exposed to chemical cues released by *Triops* had a clear disadvantage under predation by *Notonecta* compared to the morphs exposed to *Notonecta* cues. Still, compared to non-induced daphnids, they showed a limited defensive value. Surprisingly, both defended morphotypes performed equally well against *T. cancriformis*. At first glance, it seems contradictory that a mismatching defense works just as good as the specific adaptation. Even so, as two distinctive morphotypes have evolved instead of a single general defense, either the benefits or the costs (or both) have to differ in favor of the specific defense. Although the predation trials showed no direct benefits (increased survivorship), indirect benefits might exist. Such could be an increase in handling time or in predator mortality (the saw-like orientated microspines along the dorsal ridge may be able to cause injuries within *Triops’* food groove). Differences in costs are more difficult to assess, as they are often manifold [50] and depend on both abiotic and biotic factors. As such, they differ in multi-predator environments from single predator environments [48]. Depending on whether predators co-occur or occur subsequently, the costs may change even further. Therefore, it is insufficient to assess the costs of defenses by simple comparisons of predator-exposed and non-predator-exposed individuals. Predator-related environmental costs, like ‘survival trade-offs’ [48,49], can possibly surpass ‘costs of plasticity’ (that is, the costs for the ability to be plastic, for a review see [51]) by far. Costs may also be reduced under certain circumstances; for instance, when a defense against one predator simultaneously offers protection against another predator (as here in the case of *D. barbata)*. Consequently, it is crucial to understand the modalities of the predators in a given system to evaluate the costs of inducible defenses. To this end, it is helpful to visualize modality as an Euclidean vector, showing both the direction and limit of natural selection caused by a predator. Based on that, we developed a novel concept on the influence of modality in multi-predator regimes (Figure 4). In a system with one prey and two predators, three different scenarios are possible: The predators can be *functionally equivalent* (type I, Figure 4), with both vectors pointing in the same direction, *functionally inverse* (type II), with both vectors pointing in opposite directions or *functionally diverse* (type III), with both vectors pointing in different directions. Depending on the conditions, predator-specific inducible defenses can be found within each of the three categories.

Previous reports of predator specific-defenses cover either type I [3,24,36,41] or type II [48,49,52,53] but rarely type III [7,20]. Yet, systems with two predators should be most realistically described with two dimensions (type III, Figure 4). In this case, the x-axis shows phenotypic characteristics relevant for the risk caused by the first predator, while changes in the y-axis only influence the predation risk from the second. A reason for the predominance of types I and II may be a simplification by observation, which can happen if only one or a small number of related traits are observed. Then it is likely that a second predator causes selection to go in the same or the opposite direction (type I and II, respectively, Figure 4). Vice versa, with more observed traits, the chance increases to find changes relevant to one predator only (y-axis, type III, see Figure 4). Additionally, natural selection can also lead to a simplification when fitness trade-offs do not exist and predators always co-occur. Selection should then favor that type IIIa and b systems convert into type Ib, and thus display only one general defense (compare also case 2 for type IIIb, Figure 4). The same should happen if the cost of differentiating between predators is higher than the benefit of a predator-specific response. Since *D. barbata* does not display a general defense, acting against both predators, this suggests that *Triops* and *Notonecta* have a different spatial or temporal occurrence. *D. barbata* is known to inhabit both small temporary ponds and larger lakes in Africa [44,54] as does *Notonecta*[45,55,56], while *Triops* lives in temporary freshwaters as pioneer species [46,57]. Therefore, habitats might exist with only one of these two predators. An alternative explanation is that the different plastic defenses are an adaptation to a common succession pattern. When a dry pond gets filled with water, daphnids and *Triops* hatch from resting eggs. Thus, while there is an immediate threat caused by *Triops*, *Notonecta* have to migrate to the pond [55] and lay their eggs. Adult *Notonecta* occur in smaller numbers, have a reduced feeding rate (1/8 to 1/4 of earlier instars), consume more surface prey than juveniles [58,59] and, therefore, exert less predation impact on pelagic organisms such as *Daphnia*. As soon as juvenile *Notonecta* hatch they are in high numbers and represent an immediate and strong threat to *D. barbata*. By then, the daphnids should already possess their defenses (from reacting to the chemical cues of the adult notonectids), being now well adapted.

Further experiments are needed to analyze the response of *D. barbata* exposed to both predators simultaneously. Previous studies (for a review see [60]) showed that responses to two different predators usually result in an intermediate response or a response identical to the ‘more risky’ predator. However, it is just as important to acquire field data about the predator-regimes that *D. barbata* faces. Especially, as it is a condition for the two specialized defenses to evolve that the composition of the predator-regimes changes. For that predator succession seems to be the most plausible explanation. That predator succession influences the expression of inducible defenses is already known for frogs [61], but not for any daphnid species so far. The importance of predator succession might even apply to many other prey organisms as well, not only in temporary habitats, but also due to seasonal changes in temperate zones. According to our concept (see details for case IIIb, Figure 4 and Additional file 1: Figure S1), these frequently changing environments would allow for the persistence of type III systems. However, even then it is a basic condition for type III, that the predators show qualitative differences in their selection pressure. If the predators belong to different main types (true predators, grazers, parasites, parasitoids [62]) these differences might be more likely, but this is not the case for *Triops* and *Notonecta*. Thus, whether or not predators exert different selection pressures on their prey can only be answered by looking directly and in detail at the species in question.

## Conclusion

In the case of *D. barbata*, it is evident that even the modality differences of two invertebrate predators matter. This led to the ability to react to *Triops* and *Notonecta* with a wide array of distinctive and specific morphological defenses, making *D. barbata* the morphologically most plastic daphnid based on current knowledge. With all the advantages that have established *Daphnia* as model organisms, including a sophisticated genetic background [63], we hope that this study provides an experimental basis for future research and further insight into the ultimate causes for the evolution of inducible defenses. From a theoretical perspective, we hope our concept proves to be a useful extension of the four prerequisites for the evolution of inducible defenses, outlined by Tollrian and Harvell [9]. Furthermore, our concept can be easily adapted to any number of predators by using combinations of the three categories, their subgroups and, if necessary, by the addition of more dimensions. In conclusion, our study highlights the need to include predator modality in research regarding inducible defenses and predator-prey interactions in general.

## Methods

### General procedure

We used an Ethiopian clone (Eth 1) of *D. barbata*, provided by Joachim Mergeay. Of the predators used, *T. cancriformis* derived from a clonal line provided by the University of Vienna (Dr. E. Eder), while adult *N. glauca* were caught in the field and treated against bacteria and fungi (TetraMedica General Tonic, Tetra GmbH, Melle, Germany) prior to the experiments. Juvenile notonectids were obtained by hatching the adults’ eggs. Three stable laboratory cultures of *D. barbata* (beaker-set A) for all three treatments were established, starting with 13 adult, pre-induced (*Triops* or *Notonecta*) or control daphnids, which were each put in a 1.5 L beaker containing semi-artificial medium [64]. In each beaker, a 125 μm mesh net-cage was placed, which was either empty (control), or contained a single predator (*Triops* or *Notonecta*). The daphnids were fed daily with 1 mg C/l of green algae (*Scenedesmus obliquus*) and 50% of the medium was exchanged every five days. Each predator was fed 5 to 10 adult *D. barbata* and 3 live chironomid larvae per day, which were also placed in the control treatment. Impurities and feces were removed every other day. After obtaining a stable population of more than 100 daphnids in each beaker, a batch of juveniles was randomly removed once a week and put into fresh beakers (beaker-set B), which were treated in the same way as the corresponding beaker-set A and considered as biological replicates. All beakers (set A, set B and the predation trials) were kept in a climate-controlled chamber at 20 ± 0.5°C under a constant period of fluorescent light (15 h day:9 h night). Beaker-set B was checked daily for primiparous daphnids, which were then removed and counted. If a beaker contained at least 11 primiparous daphnids, 10 randomly chosen (or decimal multiples) were used in the predation trials and the rest were preserved in 70% EtOH (p.a.) for later measurements of morphological traits. If a beaker did not contain at least 11 primiparous daphnids or if not enough daphnids from another treatment were available (as each predation trial consisted of 20 daphnids, 10 from one, 10 from another morphotype), then the replicate could not be used in the predation trials and was excluded from analysis. This resulted in a total number of 21 *Triops*-induced (131 measured daphnids) and 17 control and *Notonecta*-induced replicates (control 110 and *Notonecta*-induced 95 measured daphnids).

### Measurements

Using a digital image analysis system (cell^P software and Altra 20 Camera, Olympus, Hamburg, Germany) mounted on a stereo microscope (Olympus SZX12), the following parameters were measured from a lateral view:

– body length, defined as the distance between the tail-spine base and the upper edge of the compound eye;

– helmet length, defined as the distance between the edge of the compound eye and the tip of the helmet;

– helmet angle, defined as the angle enclosed between tail-spine base, center of the compound eye and tip of the helmet;

– absolute spine length, defined as the ventral edge of the tail-spine, measured from the base to the tip using a polygon line with at least five points;

– effective spine length, defined as the straight distance between base and tip of the tail-spine;

– spine angle, defined as the angle enclosed by the tip of the tail-spine, the base of the tail-spine and the center of the compound eye.

Four additional parameters were measured from a dorsal view of the head:

– distance between the 1^st^ and the 10th dorsal spine, as a measurement of microspine density;

– maximum dorsal spine length;

– maximum dorsal ridge width;

– angle of the fifth dorsal spine relative to the dorsal ridge.

From the ratio between absolute and effective tail-spine length, another parameter, “curvature”, was calculated. To exclude body-size effects, relative values of helmet length, body width and tail-spine length were calculated. For each replicate the arithmetic mean of each trait was calculated from the single measurements and then analyzed statistically. Since the assumptions for parametric tests were not met (normal distribution and/or homogeneity of variance), Kruskal-Wallis one-way analysis of variance was performed using IBM SPSS 20.0 (IBM, Armonk, New York, USA).

### Predation experiment

Predation trials were conducted under fluorescent light in a climate chamber at 20+/−0.5°C. Each morph was tested against the others (*Notonecta* induced/control, *Triops* induced/control, *Notonecta* induced/*Triops* induced) with either *Notonecta* or *Triops* as the predator. Ten female primiparous daphnids of both respective morphs were placed into an 800 ml beaker, containing 200 ml medium. The trial started when the predator/s (one *Triops*, sized 20 to 30 mm, or three 2nd to 3rd instar *Notonecta*s, 3 to 5 mm) were placed into the beaker and ended after 90 minutes (*Triops*) or 3 hours (*Notonecta*), or when half of the daphnids were eaten. Numbers of surviving daphnids were subsequently counted using a stereo microscope (Leica MS5, Leica Microsystems, Wetzlar, Germany, 6.3× magnification). All combinations of treatments and predators was replicated eight times and analyzed with a Wilcoxon signed-rank test using IBM SPSS 20.0 (IBM, Armonk, New York, USA).

## Competing interests

The authors declare that they have no competing interests.

## Authors’ contributions

QH and CL designed the experiment. QH conducted the experiment, analyzed data and developed the concept. CL provided methods and materials. QH wrote the first draft of the manuscript and CL contributed substantially to revisions. Both authors read and approved the final manuscript.

## Supplementary Material

Additional file 1: Figure S1. Full concept for the role of modality in systems with two predators. For detailed description see Figure 4. In addition to Figure 4, optimal responses and maladaption costs of mismatching phenotypes in environments with predator succession and predator co-occurrence are given for each subgroup. Click here for file
