# Supplementary figures and images for: Modality matters for the expression of inducible defenses: introducing a concept of predator modality
Source: BMC Biol. 2013 Nov 18;11:113. doi: 10.1186/1741-7007-11-113 (PMC4225664; doi:10.1186/1741-7007-11-113)

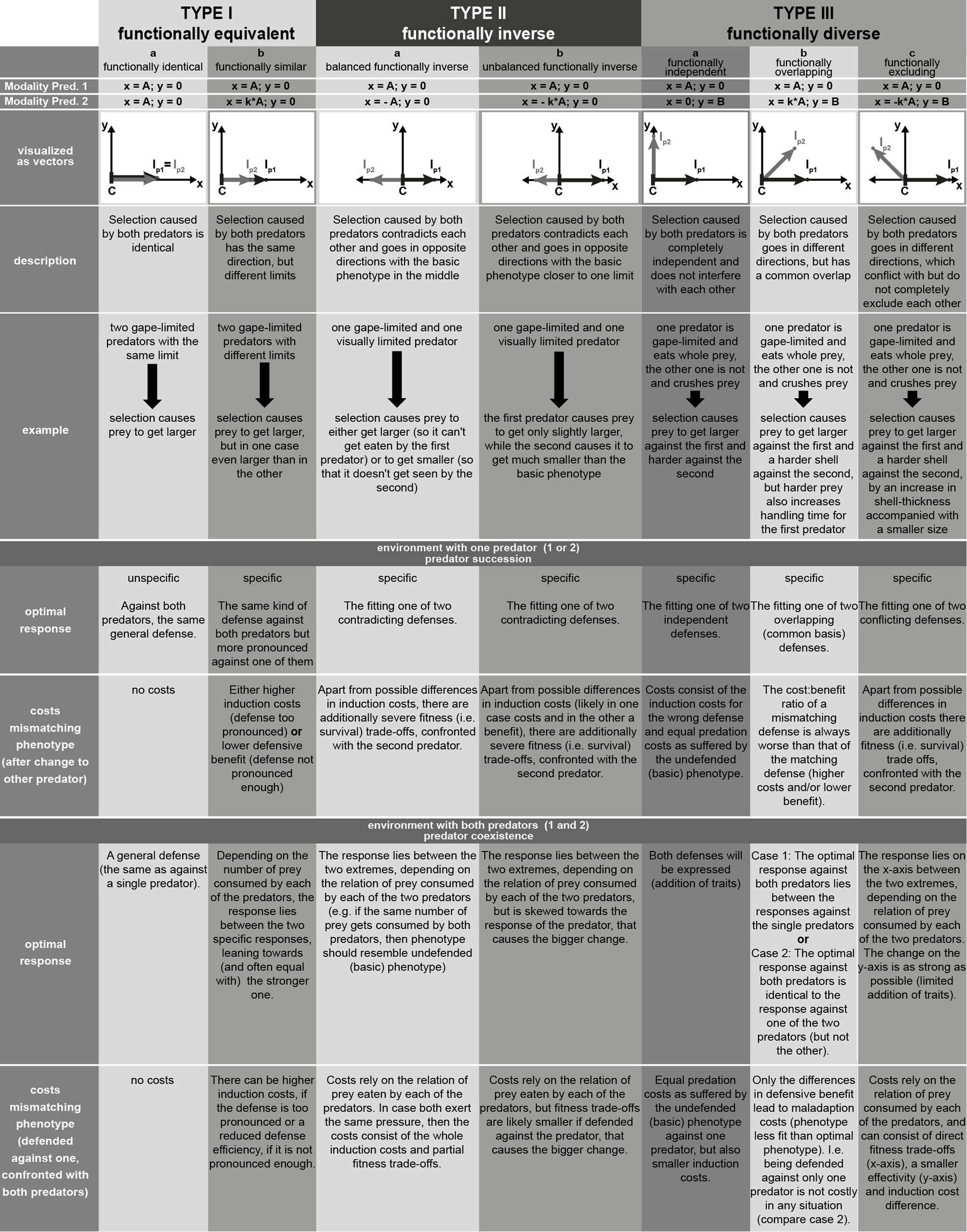

Supplement: Additional file 1: Figure S1. — Full concept for the role of modality in systems with two predators. For detailed description see Figure 4. In addition to Figure 4, optimal responses and maladaption costs of mismatching phenotypes in environments with predator succession and predator co-occurrence are given for each subgroup. [file 1741-7007-11-113-S1.tiff]
